# Weighted Gene Co-Expression Network Analysis and Alternative Splicing Analysis Reveal Key Genes Regulating Overfeeding-Induced Fatty Liver in Lion-Head Goose

**DOI:** 10.3390/ijms27010407

**Published:** 2025-12-30

**Authors:** Jing Fu, Yezhi Lan, Yuwen Liang, Xiaoguang Yang, Ruize Tang, Yuchuan Wang, Yabiao Luo, Chunpeng Liu

**Affiliations:** 1 College of Animal Science and Technology, Zhongkai University of Agriculture and Engineering, Guangzhou 510225, China; fujing@zhku.edu.cn (J.F.); liangyuwen0726@126.com (Y.L.); xiaoguangyang20@163.com (X.Y.); 15046398139@163.com (R.T.); 17825909156@163.com (Y.W.); 2 Sanya Institute, China Agricultural University, Sanya 572025, China; lanyezhi@cau.edu.cn; 3 State Key Laboratory of Animal Biotech Breeding, National Engineering Laboratory for Animal Breeding, MOA Key Laboratory of Animal Genetics and Breeding, College of Animal Science and Technology, Frontiers Science Center for Molecular Design Breeding, Beijing Key Laboratory for Animal Genetic Improvement, China Agricultural University, Beijing 100193, China; 4 College of Animal Science and Technology, Anhui Agricultural University, Hefei 230036, China

**Keywords:** Lion-head goose, fatty liver, overfeeding, WGCNA, alternative splicing

## Abstract

Lion-head goose is a large-sized breed native to Guangdong Province, China, exhibits remarkable capacity for fatty liver production under overfeeding conditions and is highly valued by local farmers and consumers. However, the molecular mechanisms driving fatty liver development in this breed are still unknown. In this study, we evaluated liver weight differences between normally fed and overfed Lion-head geese and further examined sex-specific differences following overfeeding. Overfeeding significantly increased liver weight more than 340%, and males possess a stronger capacity for lipid deposition under the same feeding regimen compared with females. RNA-Seq analysis identified 1476 differentially expressed genes (DEGs) shared by both sexes, which were mainly enriched in lipid and energy metabolism, oxidative stress, and mitochondrial pathways. In addition, 627 male-specific and 420 female-specific DEGs revealed sex-dependent differences, with males showing stronger transcriptional regulation and females exhibiting enhanced antioxidant and detoxification responses. Weighted gene co-expression network analysis (WGCNA) revealed 320 co-hub genes enriched in lipid and energy metabolism in overfeeding-induced fatty liver, along with 9 co-hub genes related to sex differences. Alternative splicing (AS) analysis detected 131 differentially spliced genes (DSGs). Integration of both approaches identified 7 overlapping genes, *HYCC2* (Hyccin PI4KA lipid kinase complex subunit 2), *AGL* (Amylo-Alpha-1,6-Glucosidase and 4-Alpha-Glucanotransferase), *CCDC62* (Coiled-coil domain containing 62), *IGSF5* (Immunoglobulin superfamily member 5), *MGARP* (Mitochondria-localized glutamic acid-rich protein), *CD80* (Cluster of Differentiation 80), and *FPGS* (Folylpolyglutamate synthase), as potential key regulators. These findings provide new insights into transcriptional and post-transcriptional regulation of overfeeding-induced fatty liver in geese.

## 1. Introduction

The liver is the primary site for lipid synthesis in birds. Under natural conditions, hepatic steatosis develops in wild Palmipeds for energy storage prior to migration. This physiological capacity is widely used in poultry production for the commercial production of fatty liver (“foie gras”) [1]. Goose fatty liver represents a physiological form of hepatic steatosis, which can increase to a weight eight to ten times heavier than normal liver after overfeeding without showing obvious pathological symptoms, suggesting that goose liver may have developed some unique protective mechanisms during long-term biological evolution [2]. Elucidating these mechanisms may help to enhance the efficiency of goose fatty liver production, and provide reference for solving the problems of Metabolic dysfunction-associated steatotic liver disease (MASLD) in humans and animals.

The Lion-head goose originates from Raoping County, Guangdong Province, China, and is mainly distributed in the Chaoshan region [3]. As the largest goose species in China, it is famous for its fast-growing, rich muscles, early sexual maturity, excellent roughage resistance [4], and excellent production performance for fatty liver [5]. Given its remarkable ability to develop fatty liver under overfeeding conditions, the Lion-head goose provides a valuable model for studying the genetic and physiological mechanisms of fatty liver formation. Males are more susceptible than (premenopausal) females to diet-induced liver fat accumulation and progression to MASLD, liver fibrosis, and hepatocellular carcinoma, in both humans and rodents [6,7]. In geese, sex-related differences are also observed in the development of liver fat accumulation [8]. These consistent sex-dependent patterns strongly imply that intrinsic regulatory factors, particularly genetic mechanisms, may shape the differential hepatic responses to overfeeding. Therefore, understanding the genetic basis is essential for elucidating these differences. However, the genetic mechanisms underlying overfeeding-induced fatty liver formation in geese, as well as the genetic basis of sex-specific differences in this process, remain unclear.

RNA sequencing (RNA-Seq) has been widely used to identify genes associated with many important traits in geese [9,10]. Weighted gene co-expression network analysis (WGCNA) enables the integration of gene expression data with phenotypic information to identify highly correlated gene modules and hub genes associated with fatty liver formation in geese. In addition to identifying differentially expressed genes (DEGs), RNA-Seq can also capture alternative splicing (AS) events, revealing the complexity of post-transcriptional regulation [11]. AS is regarded as a key factor of the increased diversity and complexity of cells and functions in higher eukaryotes [12,13]. In the mammalian genome, more than 95% of multi-exon genes are alternatively spliced to form multiple transcripts [14]. Previous studies have shown that AS-driven functional diversity of lipid metabolism-related genes may play an important role in the formation of fatty liver in geese. However, research on the diversity of mRNA alternative splicing in Lion-head goose and its potential mechanisms underlying overfeeding-induced fatty liver remains limited.

In this study, we investigated the phenotypic differences in liver weight between normally fed and overfed geese, as well as between male and female geese after overfeeding. Transcriptome sequencing of liver tissues revealed that the shared DEGs between sexes were mainly enriched in pathways related to lipid and energy metabolism, oxidative stress, and mitochondrial function. Through integrated WGCNA and AS analysis, seven potential key genes associated with overfeeding-induced fatty liver formation in Lion-head goose were identified, including *HYCC2*, *AGL*, *CCDC62*, *IGSF5*, *MGARP*, *CD80*, and *FPGS*. These findings provide new insights into the transcriptional regulatory mechanisms underlying liver hypertrophy in overfed Lion-head goose and lay a foundation for further genetic improvement and application of this valuable breed.

## 2. Results

### 2.1. Overfeeding Induces Sex-Specific Liver Hypertrophy

**Figure 1:**
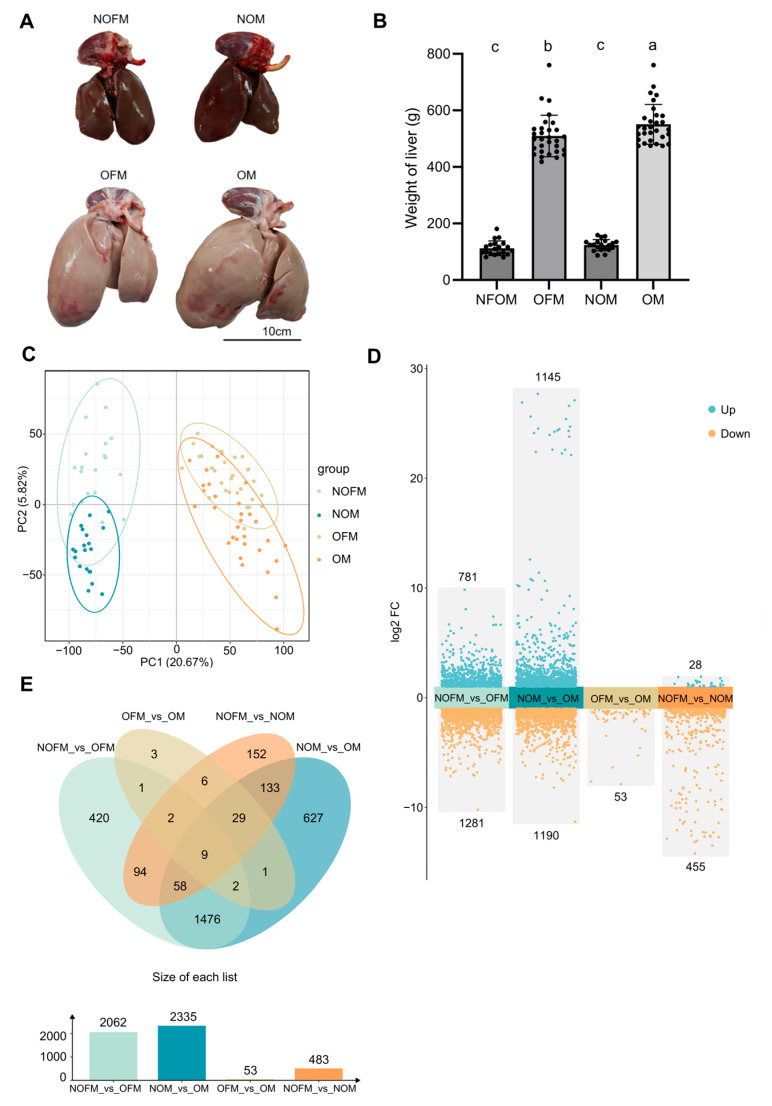
Goose liver phenotype differences between overfed and normally fed geese and between sexes. (**A**) Liver morphology in NOFM, NOM, OFM, and OM geese; (**B**) Liver weight comparison among the four groups. Different lowercase letters (a–c) above the bars indicate significant differences among groups (one-way ANOVA followed by Tukey’s post-hoc test, *p* < 0.05). Letters are assigned based on the group means from highest (a) to lowest (c), and bars sharing the same letter do not differ significantly. Identification of DEGs between overfed and normally fed geese and between sexes. (**C**) Partial Least Squares Discriminant Analysis (PLS-DA) of transcriptome profiles among the NOFM, NOM, OFM, and OM groups. The *X*-axis (PC1) and *Y*-axis (PC2) represent the first and second latent variables extracted by PLS-DA, which capture the greatest variation relevant to group discrimination; (**D**) The numbers of upregulated and downregulated DEGs from four comparisons; *p* values for DEGs selecting were adjusted using the Benjamini–Hochberg method. (**E**) Venn–bar plot of shared and sex-specific DEGs.

Phenotypic measurements showed that overfeeding resulted in a significant enlargement of the liver in both male and female Lion-head goose (Figure 1A). Liver color was visually assessed and showed an apparent change from dark red to light pink following overfeeding. Further liver weight analysis revealed that before overfeeding, the average liver weights of male and female geese were 123.55 ± 19.73 g and 112.40 ± 25.77 g, respectively, with no significant difference. After overfeeding, the liver weights increased to 550.93 ± 70.32 g in males and 509.73 ± 73.23 g in females, with the male geese exhibiting significantly heavier livers than females (Figure 1B). These results indicate that male Lion-head geese possess a stronger capacity for lipid deposition under overfeeding conditions compared with females.

### 2.2. Identification of RNA-Seq Data

Liver tissue samples were sequenced with 30 replicates in each overfed group and 20 replicates in each normally fed group. After quality control, the range of clean data was between 40,417,180 and 64,117,456 bp. The proportions of Q20 and Q30 were greater than 95.67% and 90.16%, respectively, and the GC content ranged from 43.41% to 47.57%. More than 93.29% of reads were mapped to the reference genome (Table S3). To assess differences between groups, PLS-DA analysis was performed on the fragments per kilobase of transcript per million mapped reads (FPKM) values of all samples (Figure 1C).

### 2.3. Analysis of DEGs Between Overfed and Normally Fed Geese and Between Sexes

FPKM values were used to quantify gene and transcript expression across four goose groups differing in feeding treatment or sex. DEGs were identified with a threshold of |log_2_FoldChange| ≥ 1 and *p* ≤ 0.05. Comparisons among the four groups and obtained 2062 DEGs (NOFM vs. OFM), 2335 (NOM vs. OM), 53 (OFM vs. OM) and 483 (NOFM vs. NOM), respectively. Among these, the numbers of upregulated and downregulated genes were 781/1282; 1145/1190; 0/53; and 28/455 (Figure 1D). For each comparison, the top ten most highly upregulated and downregulated genes are summarized in Table S4. In total, 1476 co-expressed DEGs (co-DEGs) related to fatty liver traits were identified, showing consistent differential expression between overfed and normally fed geese in both male and female geese. Additionally, 627 DEGs were specifically found in male geese and 420 in female geese (Figure 1E).

### 2.4. GO and KEGG Functional Annotation of Co-DEGs and Sex-Specific DEGs

**Figure 2:**
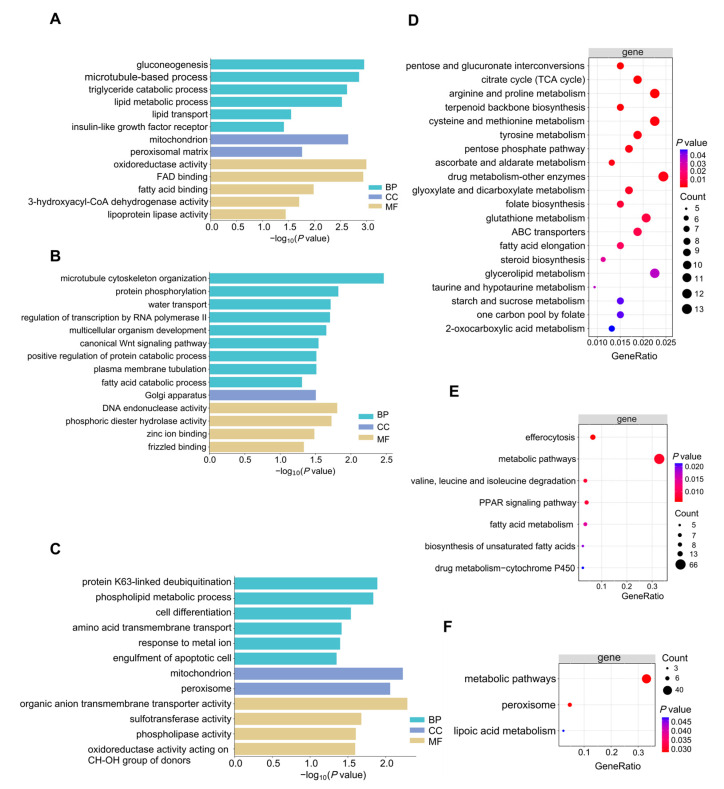
GO and KEGG functional annotation of co-DEGs and sex-specific DEGs. (**A**) GO terms of 1476 shared DEGs; (**B**) GO terms of 627 male-specific DEGs; (**C**) GO terms of 420 female-specific DEGs, the genes involved in the major GO biological process presented in Figure 2A–C are summarized in Table S5; (**D**) KEGG pathways of shared DEGs; (**E**) KEGG pathways of male-specific DEGs; (**F**) KEGG pathways of female-specific DEGs. The size of the bubbles is proportional to gene counts; the legend shows representative sizes, not exact values for each bubble. BP: Biological Process; CC: Cellular Component; MF: Molecular Function. *p* values were adjusted using the Benjamini–Hochberg method.

GO enrichment analysis revealed that the 1476 co-DEGs, consistently identified in both overfed males (OFM vs. NOM) and overfed females (OFF vs. NOFM), were mainly involved in energy metabolism, lipid metabolism, and redox-related biological processes, indicating common molecular responses to overfeeding-induced fatty liver formation in both sexes (Figure 2A). The significantly enriched terms of the 627 male-specific DEGs (NOM vs. OM) and the 420 female-specific DEGs (NOFM vs. OFM) highlighted distinct sex-related biological distinctions. Male-specific DEGs (NOM vs. OM) were predominantly enriched in regulatory and signaling-related processes (Figure 2B). However, female-specific DEGs (NOFM vs. OFM) were mainly associated with transport, detoxification, and redox-related functions (Figure 2C).

KEGG pathway enrichment analysis further supported these observations, showing that the 1476 co-DEGs were significantly enriched in pathways related to central carbon metabolism as well as lipid metabolism and antioxidant processes, reflecting enhanced energy production and biosynthetic capacity, together with coordinated regulation of lipid accumulation and oxidative stress responses in both sexes under overfeeding conditions (Figure 2D). Male-specific DEGs (NOM vs. OM) were preferentially enriched in pathways related to lipid utilization and cellular clearance (Figure 2E), whereas female-specific DEGs (NOFM vs. OFM) were mainly enriched in peroxisome-related and antioxidant pathways (Figure 2F). Collectively, these findings suggest that male and female geese adopt distinct molecular strategies to cope with lipid accumulation and oxidative stress during overfeeding-induced fatty liver formation.

### 2.5. WGCNA-Based Identification of Hub Genes Associated with Overfeeding-Induced Fatty Liver and Sex Differences

**Figure 3:**
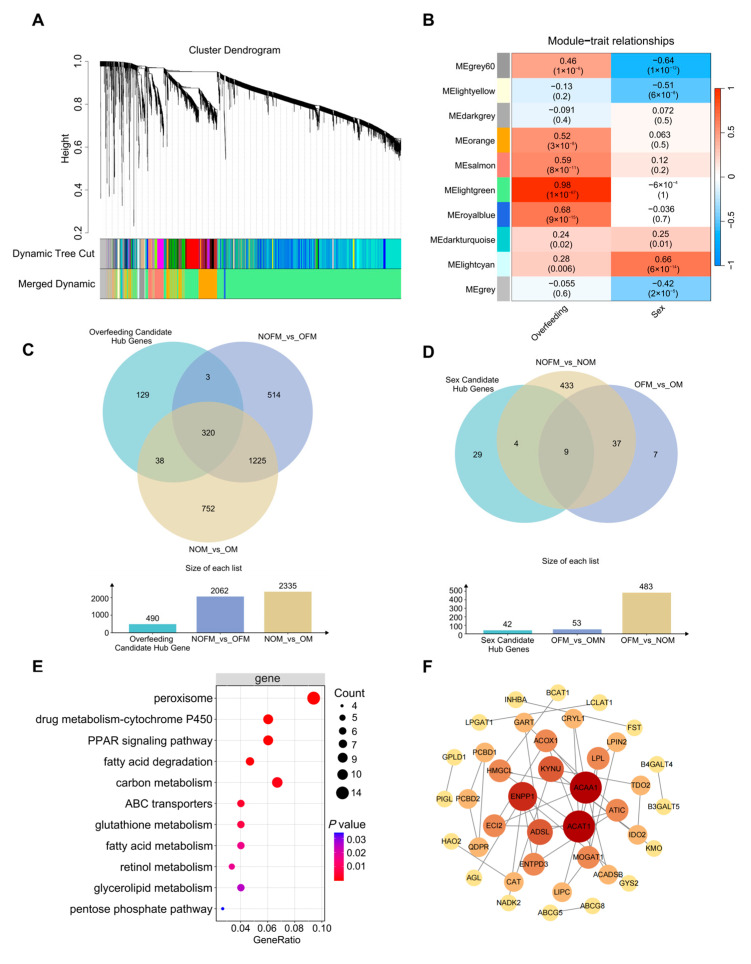
WGCNA of DEGs associated with fatty liver and sex differences. (**A**) Gene clustering dendrogram generated from topological overlap matrix. The *y*-axis represents the clustering height (dissimilarity), and branches correspond to individual genes. The color bar below shows module assignment, including the initial dynamic tree cut and the merged dynamic modules; (**B**) Module–trait correlation heatmap showing associations between module eigengenes and phenotypes, including fatty liver status and sex. Cell color indicates correlation strength (red = positive, blue = negative), and numbers show correlation coefficients (upper) and *p* values (lower); (**C**) Venn–bar plot of the intersection of fatty liver-related hub genes and DEGs; (**D**) Venn–bar plot of the intersection of sex-related hub genes and DEGs; (**E**) KEGG pathways of fatty liver-related co-hub genes, *p* values were adjusted using the Benjamini–Hochberg method; (**F**) Protein–protein interaction network of top-ranking co-hub genes. Node size scales with degree, and node color reflects centrality (darker = higher). Edges indicate predicted interactions.

To further investigate the relationship between traits and DEGs, the top 5000 genes with the highest expression variability were selected for WGCNA. The hierarchical clustering tree of gene clusters was constructed, and modules with similar expression profiles were merged, resulting in a total of 10 modules (Figure 3A). To identify key modules related to overfeeding-induced fatty liver and sex-related differences, correlations between modules and phenotypic traits were analyzed. The lightgreen (*r* = 0.98), royalblue (*r* = 0.68), salmon (*r* = 0.59), and orange (*r* = 0.52) modules showed strong correlations with fatty liver after overfeeding, respectively. The lightcyan (*r* = 0.66), grey60 (*r* = −0.64), and lightyellow (*r* = −0.51) modules were strongly associated with sex differences, respectively (Figure 3B).

Within these highly correlated modules, 490 candidate hub genes related to overfeeding-induced fatty liver and 42 candidate hub genes related to sex differences were identified. Intersection of the 490 fatty liver-related hub genes with the DEGs from the NOFM vs. OFM and NOM vs. OM comparison yielded 320 co-hub genes (Figure 3C). KEGG enrichment analysis of these genes revealed significant enrichment in pathways related to lipid metabolism, energy metabolism, drug metabolism, and oxidative stress (Figure 3E). A gene–gene interaction network was constructed the using the top-ranking co-hub genes on the basis of degree centrality (Figure 3F). Similarly, intersection of the 42 candidate sex-related hub genes with DEGs from the OFM vs. OM and NOFM vs. NOM comparisons resulted in 9 co-hub genes: *LRRC2* (Leucine rich repeat containing 2), *LOC106038083*, *LOC125179635*, *UBE2R2* (Ubiquitin conjugating enzyme E2 R2), *LOC106048542*, *LOC136788421*, *LOC136789444*, *SLC1A1* (Solute carrier family 1), and *LOC106048358* (Figure 3D).

### 2.6. Different Alternative Splicing Analysis Events of Overfeeding-Induced Fatty Liver

**Figure 4:**
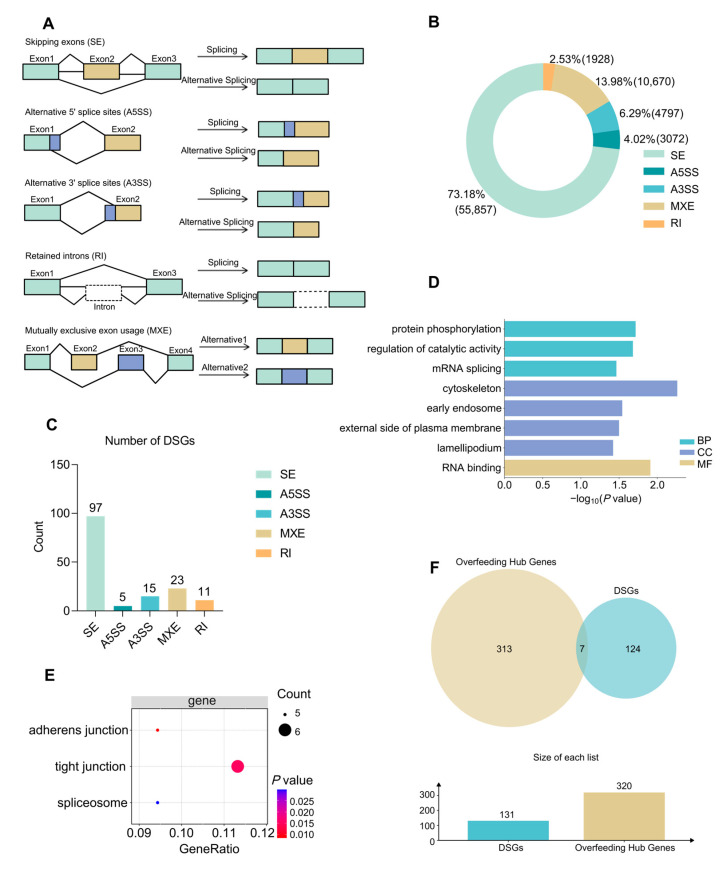
AS analysis in overfeeding-induced fatty liver. (**A**) The diagram of five types of AS events; (**B**) Proportions of the five AS event types; (**C**) Numbers of DSGs in each AS events; GO (**D**) and KEGG (**E**) enrichment analysis of DSGs. The genes involved in the major GO biological process presented in (**D**) are summarized in Table S5; *p* values were adjusted using the Benjamini–Hochberg method. (**F**) Overlap of co-hub genes and DSGs.

To focus on alternative splicing (AS) in overfeeding-induced fatty liver, we analyzed AS events between the overfed and normally fed groups. A total of 76,324 AS events were identified in both the overfed and normally fed groups. Among these, skipped exon (SE) events were the most abundant, accounting for 55,857 events (73.18%), followed by mutually exclusive exon (MXE) events (10,670; 13.98%), alternative 3′ splice site (A3SS) events (4797; 6.29%), alternative 5′ splice site (A5SS) events (3072; 4.02%), and retained intron (RI) events (1928; 2.53%) (Figure 4A,B). A total of 131 significantly differentially spliced genes (DSGs) were identified. Specifically, the numbers of DSGs associated with SE, A5SS, A3SS, MXE, and RI events were 97, 5, 15, 23, and 11, respectively (Figure 4C).

GO enrichment analysis revealed that these DSGs were mainly enriched in terms related to mRNA splicing, RNA binding, cytoskeleton, early endosome (Figure 4D). KEGG enrichment analysis revealed that these DSGs were primarily enriched in pathways related to spliceosome, tight junction and adherens junction (Figure 4E).

By intersecting the 320 co-hub genes identified from WGCNA with the 131 DSGs, a total of 7 overlapping genes were discovered, including *HYCC2*, *AGL*, *CCDC62*, *IGSF5*, *MGARP*, *CD80*, and *FPGS* (Figure 4F). These genes not only occupy central positions within the gene co-expression and interaction networks, but also exhibit significant alternative splicing events. Collectively, these findings suggesting that these seven genes may serve as key regulatory factors linking gene expression and post-transcriptional modification in the development of overfeeding-induced fatty liver in Lion-head goose.

### 2.7. Validation of DEGs and AS Events

**Figure 5:**
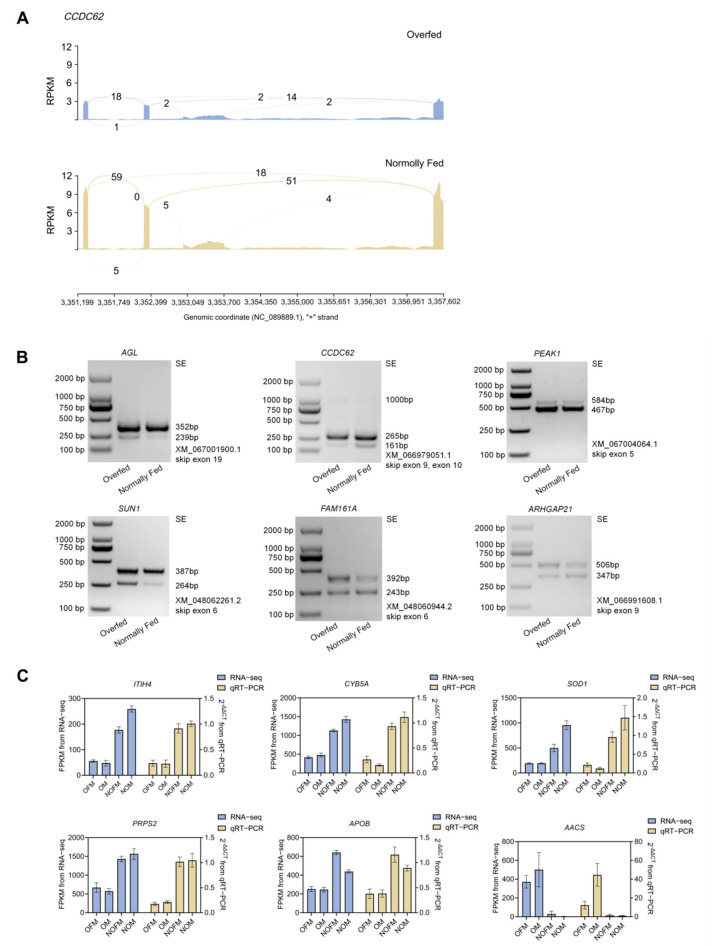
Validation of RNA-Seq and AS results. (**A**) Sashimi plots of *CCDC62* showing SE events. The *y*-axis indicates normalized read coverage (RPKM), reflecting the expression level of exonic regions, while the numbers on the arcs represent junction-spanning read counts supporting alternative splicing events. (**B**) RT-PCR validation of 6 AS events; (**C**) qPCR validation of 6 DEGs.

To verify the reliability of RNA-Seq results, six DEGs—*ITIH4* (Inter-alpha-trypsin inhibitor heavy chain 4), *CYB5A* (Cytochrome b5 type A), SOD1 (Superoxide Dismutase 1), *PRPS2* (Phosphoribosyl pyrophosphate synthetase 2), *APOB* (Apolipoprotein B), and *AACS* (Acetoacetyl-CoA synthetase)—were randomly selected for further verification by quantitative real-time PCR (qPCR). The expression patterns obtained from qPCR were consistent with the RNA-Seq results across all four groups, confirming the accuracy and reproducibility of the transcriptome data (Figure 5C). Pearson correlation analysis confirmed strong concordance between the two methods for all six genes (*AACS*: *r* = 0.7521, *p* = 0.0002; *APOB*: *r* = 0.9285, *p* < 0.0001; *CYB5A*: *r* = 0.9529, *p* < 0.0001; *ITIH4*: *r* = 0.9063, *p* < 0.0001; *PRPS2*: *r* = 0.8917, *p* < 0.0001; *SOD1*: *r* = 0.8717, *p* < 0.0001), confirming the reliability and reproducibility of the RNA-Seq data.

To validate the AS events, we selected an overlapping gene, *CCDC62*. Sashimi plots showed *CCDC62* underwent two SE events between 3,351,199 and 3,357,602, with exon 10 being skipped more frequently than exon 9. In addition, its gene expression level significantly decreased after overfeeding (Figure 5A), suggesting that *CCDC62* may play an important role in alternative splicing-mediated regulation during the development of overfeeding-induced fatty liver. Six AS events from DSGs were randomly selected for RT-PCR validation, and the results were consistent with the RNA-seq data (Figure 5B).

## 3. Discussion

The liver is the primary site of lipid synthesis in geese. Overfeeding provides an excess of energy substrates, particularly glucose, leading to enhanced fatty acid and triglyceride synthesis. When triglyceride production exceeds the transport capacity of *VLDL* (Very Low Density Lipoprotein) and the degradative capacity of β-oxidation, lipid accumulation occurs in hepatocytes [15]. Transcriptome analysis identified 1476 co-DEGs shared among four comparisons (NOFM vs. OFM, NOM vs. OM, OFM vs. OM and NOFM vs. NOM). During the development of fatty liver in geese, these genes are mainly involved in lipid metabolic processes, energy metabolism pathways (gluconeogenesis, TCA cycle, pentose phosphate pathway), and oxidative stress responses. Additionally, several hub genes identified by WGCNA, including *ACAT1* (Acetyl-CoA acetyltransferase 1), *LIPC* (Lipase C, hepatic type), *GPLD1* (Glycosylphosphatidylinositol specific phospholipase D1), *ACOX1* (Acyl-CoA oxidase 1), *LPIN2* (Lipin 2), *LPL* (Lipoprotein lipase), *ECI2* (Enoyl-CoA delta isomerase 2), and *ACAA1* (Acetyl-CoA acyltransferase 1), have been verified in other species and identified as related to fatty liver. For example, during overfeeding, *ACAT1*, *LIPC*, and *GPLD1* in Pekin, Muscovy, Mule, and Hinny ducks; *ACOX1* and *LPIN2* in Muscovy, Mule, and Hinny ducks; *LPL* and *ECI2* in Hinny ducks; and *ACAA1* in Muscovy ducks, all involved in lipid catabolic process [16]. *ACAT1* regulates glycolysis and ketogenesis, its reduction will increase the level of circulating ketones and promote hepatic steatosis [17]. *LIPC* is involved in metabolic, immune and lipid functions [18], while *GPLD1* participates in glucose and lipid homeostasis [19]. *ACOX1, LPIN2, LPL, ECI2, and ACAA1* coordinate β-oxidation and glycerolipid metabolism, highlighting conserved mechanisms across waterfowl species [20,21,22,23,24]. Insulin receptor pathways were also enriched, which reflects alterations in hepatic glucose uptake and utilization through glycolysis and lipogenesis during fatty liver development [25]. Other hub genes such as *ADSL* (Adenylosuccinate lyase) and [26] *ENPP1* (Ectonucleotide Pyrophosphatase/Phosphodiesterase 1) [27], further suggest complex interaction between insulin sensitivity, lipid accumulation, and energy homeostasis. To facilitate interpretation of the Results, the key genes identified across WGCNA, differential expression, and alternative splicing analyses discussed in this section are summarized in Table S6, and the numbers of genes obtained from each analysis method are summarized in Table S7.

Lipid accumulation is accompanied by oxidative stress and mitochondrial dysfunction, evidenced by enrichment in glutathione metabolism, oxidoreductase activity, and peroxisomal and mitochondrial functions. During the development of MASLD, excessive ROS (Reactive Oxygen Species) production leads to lipid peroxidation and impaired ATP synthesis [28]. MASLD has a high tendency to induce mitochondrial dysfunction, including reduced mitochondrial membrane potential and downregulated oxidative phosphorylation [29]. Alterations in mitochondrial membrane potential can lead to apoptosis, which in turn may be important in the progression of MASLD [30]. However, glucose can protect normal mitochondrial function by enhancing anti-oxidant capacity in goose fatty liver, thereby inhibiting apoptosis [31]. Moreover, lipids synthesized in the liver are mainly transported by *VLDL*, and dietary lipids are transported by chylomicrons [32]; if they are not hydrolyzed by *LPL* present in the blood, they will return to the liver via specific receptors, further promoting hepatic fat deposition and fatty liver formation [33]. Notably, in the term of lipoprotein lipase activity that we have enriched, the *LPL* gene was significantly downregulated, which may reduce lipid hydrolysis in the circulation and lead to more lipids being returned to the liver, thereby aggravating hepatic fat deposition. Collectively, these findings indicate a tight link between metabolic overload, oxidative stress, and mitochondrial impairment in the progression of fatty liver.

Alternative splicing represents another layer of post-transcriptional regulation. We found that SE accounted for the majority of the five main types of AS events, which is consistent with previous observations in other vertebrates [14,34]; SE may represent the most common alternative splicing pattern in vertebrates. However, its high frequency may result from both biological preference and the greater detectability of SE events by short-read RNA-seq. GO and KEGG enrichment results revealed that alternative splicing is highly active and contributes to post-transcriptional regulation and intracellular remodeling during overfeeding-induced fatty liver, including the modulation of RNA processing, cytoskeletal dynamics, and intercellular junctions. *AGL* deficiency causes glycogen storage disease type III (GSD III), which is characterized by abnormal hepatic glycogen accumulation, steatosis, and progressive fibrosis. Consistent with clinical findings that splice site variants account for a large proportion of pathogenic *AGL* mutations in GSD III [35], our results suggest that splicing alterations of *AGL* may also occur in geese. Such changes could influence the balance between glycogen mobilization and lipid conversion, thereby regulating the development of fatty liver. *MGARP* is a mitochondria-localized, glutamic acid-rich protein [36], and its overexpression alters mitochondrial structural integrity, subcellular distribution, and motility [37], which may contribute to mitochondrial dysfunction in overfeeding-induced fatty liver of geese. Alternative splicing of *FPGS* has been shown to affect drug metabolism and resistance in acute lymphoblastic leukemia (ALL) by modulating folate homeostasis [38], and since the folate has been shown to improve MASLD [39], similar splicing alterations of FPGS may also influence fatty liver development. *CCDC62* acts as a transcriptional co-activator of estrogen receptor β (ERβ), enhancing ERβ-mediated transcription and downstream gene expression [40]. ERβ agonists have been reported to exert therapeutic benefits in NASH by directly regulating the functions of xenobiotics and bile acid receptors in the liver, and exert their effects indirectly by inhibiting obesity [41]. Therefore, *CCDC62* may indirectly modulate hepatic lipid metabolism through regulation of ERβ target genes. However, direct evidence linking *CCDC62* to MASLD is currently lacking, and its hepatic function requires further investigation. *IGSF5* encodes a junctional adhesion molecule [42] localized at epithelial tight junctions and is involved in maintaining tissue integrity through interactions with scaffold proteins such as *MAGI-1* [43]. Dysregulated *IGSF5* expression has been linked to disrupted junctional organization and epithelial pathology. In this study, the identification of *IGSF5* as an alternatively spliced hub gene suggests that alterations in cell–cell junctions and hepatic structural remodeling may accompany lipid accumulation during overfeeding-induced fatty liver, rather than directly affecting lipid metabolic pathways. *CD80* is a co-stimulatory molecule regulates T-cell activation and immune homeostasis through interactions with *CD28* (CD28 molecule), *CTLA-4* (Cytotoxic T lymphocyte-associated antigen-4), and *PD-L1* (Programmed cell death 1 ligand 1) [44]. *CD80* can limit PD-1–mediated inhibitory signaling by forming cis interactions with *PD-L1* on antigen-presenting cells, while simultaneously promoting *CD28*-dependent co-stimulation [45]. Moreover, dysregulation of *CD80* expression has been linked to excessive immune activation, as increased *CD80/CD86* (Cluster of Differentiation 86) levels on macrophages and dendritic cells drive T-cell activation and inflammatory responses when immune regulatory mechanisms are impaired [46]. These findings suggest that altered *CD80* expression may contribute to immune modulation and inflammatory remodeling in the liver under metabolic stress conditions. *HYCC2* (also known as *FAM126B,* family with sequence similarity 126 member B) encodes a paralogous subunit of the PI4KIIIα lipid kinase complex, which is involved in *PI4P* (phosphatidylinositol 4-phosphate) synthesis and lipid-associated signaling pathways [47]. Although direct functional evidence for *HYCC2* remains limited, one study indicated that *HYCC2* can partially compensate for *FAM126A* deficiency and may contribute to maintaining PI4KIIIα complex stability [48], suggesting a potential role in cellular lipid organization and membrane-related processes rather than direct metabolic regulation. These findings suggest that alternative splicing in key hub genes may contribute to hepatic steatosis by potentially affecting glycogen–lipid balance, mitochondrial function, cellular structure, and immune responses.

While overfeeding induces a broadly conserved physiological response in both sexes, accumulating evidence from mammals [6,7,49,50] and geese [8,51] indicates that sex-related factors also modulate fatty liver development. Consistently, although both sexes showed marked liver enlargement after overfeeding, males possess a stronger capacity, suggesting potential sex-dependent differences in the capacity to cope with excessive lipid influx. While most DEGs were shared between sexes, reflecting a largely conserved molecular foundation, a smaller part of sex-specific DEGs indicated divergent regulatory adaptations underlying distinct physiological responses. Male geese mobilized a larger number of DEGs in response to overfeeding and showed enrichment of pathways related to transcriptional regulation, lipid metabolism, and cellular clearance. Notably, canonical Wnt signaling, branched-chain amino acid (BCAA) degradation, and efferocytosis were prominent. Wnt/β-catenin signaling plays essential roles in liver development, regeneration, and metabolic homeostasis [52], and has been recognized as a key regulator of adipose differentiation and exerts anti-lipid formation and anti-inflammatory effects [53]. BCAAs, including leucine, isoleucine (Ile), and valine (Val), can regulate fatty acid synthesis, transport, oxidation, lipolysis, and adipokine secretion depending on varying ratios [54], and circulating BCAA levels show sex-dependent associations with MASLD severity [55]. In addition, efferocytosis facilitates the clearance of dying cells and promotes pro-resolving macrophage signaling, thereby supporting recovery from liver injury induced by metabolic stress [56]. In contrast, female geese exhibited enrichment of pathways associated with sulfotransferase activity, K63-linked deubiquitination, and lipoic acid metabolism, suggesting an adaptive strategy emphasizing antioxidant defense and metabolic detoxification. Sulfotransferases are major phase II drug-metabolizing enzymes [57], and widely distributed in the liver [58]. This enzyme family sulfates various endogenous and exogenous molecules [59]; for example, *SULT2B1b* (Sulfotransferase Family 2B Member 1) mediates oxysterol sulfation to suppress LXR-SREBP-1c signaling, thereby reducing lipid accumulation in the liver [60]. *SULT1A1* (Sulfotransferase family 1A member 1) plays critical roles in xenobiotic detoxification, and its dysregulation has been linked to impaired hepatic clearance capacity in MASLD progression [57]. K63-linked deubiquitination regulates antioxidant responses mainly through the Keap1–NRF2 axis, enhancing cellular resistance to oxidative stress [61,62]. Moreover, lipoic acid serves as a crucial cofactor for mitochondrial dehydrogenases, sustaining energy metabolism and redox balance [63]. Our findings indicate that male and female geese adopt different adaptive strategies to deal with overfeeding-induced fatty liver. Males seem to rely more on enhancing transcriptional activity, lipid metabolism, and cellular clearance, whereas females appear to emphasize antioxidant defense and metabolic detoxification.

In addition, several hub genes identified by WGCNA overlapped with candidate sex-related DEGs, suggesting potential roles in sex-specific regulation. *LRRC2* is a mitochondrially relevant protein regulated by PGC-1α [64] and may be involved in hepatic mitochondrial adaptation to lipid overload. *UBE2R2* is an enzyme involved in ubiquitination with female-biased expression reported in Muscovy duck gonads before and after sex differentiation [65], and it may contribute to sex-dependent stress responses through ubiquitin-mediated protein turnover. *SLC1A1* encodes a glutamate/cysteine transporter, which regulates glutamatergic transmission and supports glutathione synthesis for antioxidant defense [66]. Although these genes are not directly involved in lipid catabolism, they may modulate mitochondrial function, redox homeostasis, and cellular stress responses, thereby contributing to sexual dimorphism in hepatic adaptation to overfeeding.

Although this study was conducted specifically in Lion-head geese, several of the molecular responses observed here, including transcriptomic changes and alternative splicing patterns associated with overfeeding-induced fatty liver, are consistent with mechanisms reported in other avian species and mammalian models of hepatic steatosis. This suggests that certain regulatory features identified in this study may reflect conserved responses to excessive nutrient intake. However, differences in genetic background, metabolic characteristics, and feeding strategies among goose breeds and across species should be considered. Particularly, potential breed differences in lipid metabolism and tolerance to hepatic lipid accumulation may influence the magnitude and timing of acute responses to overfeeding. Therefore, while our findings provide useful insights into the molecular basis of fatty liver development, their extrapolation to other goose breeds or laboratory animals should be conducted with caution and requires further validation in additional models.

It should also be noted that the 24 h protocol represents a short-term overfeeding model designed to capture early molecular and metabolic responses to excessive nutrient intake. While the overfeeding model effectively induces hepatic steatosis in geese, it mainly reflects an acute metabolic response and may not fully recapitulate the chronic pathological progression of long-term fatty liver disease. In addition, individual body weight measurements before and after the overfeeding period were not recorded, which limited a more comprehensive assessment of whole-body metabolic changes associated with the treatment. Nevertheless, liver weight and morphological traits—direct indicators of hepatic steatosis—were systematically measured and consistently reflected the physiological impact of overfeeding. Moreover, although transcriptomic and alternative splicing analyses provided insights into gene regulatory changes, the lack of metabolomic or proteomic data limited direct interpretation of downstream metabolic fluxes and lipid intermediates. Moreover, the functions of identified hub genes and splicing variants were inferred primarily from bioinformatic analyses and published evidence, without experimental validation through targeted functional assays such as gene manipulation or enzymatic activity measurements. Future studies integrating multi-omics approaches and functional experiments will be essential to validate these regulatory mechanisms and clarify their causal roles in fatty liver development.

## 4. Materials and Methods

### 4.1. Animals and Sample Collection

This study was designed as a controlled animal experiment with a two-factor design (feeding regime × sex) to investigate transcriptomic and post-transcriptional regulation associated with overfeeding-induced fatty liver in Lion-head geese. This experimental design employed a short-term (24 h) overfeeding model aimed at capturing early molecular and metabolic responses associated with hepatic lipid accumulation. The Ministry of Agriculture of China’s Guidelines for the Care and Use of Experimental Animals were strictly adhered to during all animal procedures. A total of 100 Lion-head geese, comprising 50 male geese and 50 female geese, were obtained from the Lixing Lion-head Goose Breeding Base in Chaozhou, Guangdong Province. All geese were 116 days old at the start of the study. The geese were randomly divided into the overfed group (female: *n* = 30; male: *n* = 30) and the normally fed group (female: *n* = 20; male: *n* = 20). The geese in the overfed group were force-fed with cooked brown rice for 24 h prior to sacrifice, with each goose receiving 150 g of cooked brown rice every 2 h; water was provided ad libitum throughout the overfeeding period. In contrast, geese in the normally fed group were allowed free access to cooked brown rice and water [5]. Twenty-four hours later, the geese were humanely slaughtered. Liver tissues were immediately excised and weighed using an electronic scale (Puchun, Shanghai, China), and liver samples were placed on a standardized 40 cm × 30 cm measurement board (Qingyi, Huizhou, China) and photographed using an iPhone 15 under natural light conditions to preserve the original color and morphological features of the liver. Statistical significance was determined using one-way ANOVA. As liver samples were processed and sequenced under identical conditions within a single batch, batch effects were considered negligible and were not further corrected. All samples were collected, transported on dry ice and stored at −80 °C until RNA sequencing was carried out.

### 4.2. RNA Extraction, Library Construction, and DNBSEQ High-Throughput Sequencing

Approximately 50 mg of frozen liver tissue was homogenized in 1 mL TRNzol Universal RNA Reagent (TIANGEN, Beijing, China) under RNase-free conditions using a mechanical tissue homogenizer (Retsch, Haan, Germany). Total RNA was extracted according to the manufacturer’s instructions. RNA purity and concentration were assessed using a NanoDrop 2000 spectrophotometer (Thermo Fisher Scientific, St. Louis, MO, USA), and RNA concentration and total amount were accurately quantified using a Qubit fluorometer. The size distribution of RNA fragments and RNA integrity were evaluated by agarose gel electrophoresis. mRNA was enriched using oligo (dT) magnetic beads (mRNA Capture Beads) and then fragmented. First- and second-strand cDNA synthesis was performed to obtain double-stranded cDNA (ds cDNA), followed by end repair and addition of an A-tail (dA-tailing). Sequencing adapters were ligated to the cDNA fragments, and the ligated products were purified or size-selected using magnetic beads. PCR amplification was carried out according to experimental requirements, and the concentration and size distribution of the amplified products were assessed. The PCR products were then denatured and circularized, and linear molecules were digested with exonucleases. Finally, the libraries were pooled according to the desired sequencing depth and sequenced on the DNBSEQ high-throughput sequencing platform.

### 4.3. Reads Quality Control and Mapping

FastQC (v0.12.1) was used to calculate GC content, Q20 and Q30. Clean reads were aligned to the *Anser cygnoides* T2T reference genome [67] (assembly: Taihu_goose_T2T_genome, NCBI accession no. GCF_040182565.1) in orientation mode using HISAT2 (v 2.2.1) [68], and the resulting alignment files were saved in SAM format. The SAM files were then converted to BAM format using Samtools (v1.19.2) [69].

### 4.4. Differential Expression Analysis

To identify the DEGs between different groups, the expression level of each gene was calculated using the fragments per kilobase of transcript per million mapped reads method. The DESeq2 (v1.22.1) [70] was used to conduct differential expression analysis. DEGs were defined based on |log2FoldChange| ≥ 1 and *p* values < 0.05. We used “OM”, “OFM”, “NOM” and “NOFM” to represent “overfed male group geese”, “overfed female group geese”, “normally fed male group geese” and “normally fed female group geese”, respectively.

### 4.5. Alternative Splicing Analysis

Alternative splicing (AS) events between the treatment and control groups were identified using the rMATS (v4.0.2) [21]. rMATS is capable of detecting five major types of AS events: skipped exons (SEs), mutually exclusive exons (MXEs), alternative 5′ splice sites (A5SSs), alternative 3′ splice sites (A3SSs), and retained introns (RIs). Differential alternative splicing (DAS) events were identified based on |IncLevelDifference|> 0.1, *p* < 0.05, and representative DAS events were visualized using Sashimi plots generated with rmats2sashimiplot (v2.0.4).

### 4.6. Functional Enrichment

Gene ontology (GO) functional enrichment and KEGG pathway analyses were performed using DAVID (NCBI, https://davidbioinformatics.nih.gov/ accessed on 18 June 2025). The results of the GO and KEGG analyses were visualized using the Bioinformatics online platform (https://www.bioinformatics.com.cn/ accessed on 18 June 2025).

### 4.7. Weighted Gene Co-Expression Network Analysis (WGCNA)

To identify genes that play critical roles in overfeeding-induced fatty liver and sex-related differences, a gene co-expression network was constructed using the WGCNA (v1.73) package in R (v4.5.0) [71]. Pairwise Pearson correlation coefficients were calculated between all genes to generate a correlation matrix. The soft-thresholding power was selected by evaluating scale-free topology criteria, and β = 9 was chosen as the lowest value achieving R^2^ ≥ 0.80 (Figure S1), and a weighted adjacency matrix was constructed accordingly. This matrix was then transformed into a topological overlap matrix (TOM) based on expression similarity, followed by hierarchical clustering of genes based on TOM dissimilarity. Gene modules were identified using the dynamic tree cut algorithm, and similar modules were subsequently merged. Module eigengenes were correlated with phenotypic traits to identify modules significantly associated with the traits of interest. Within the significant modules, candidate hub genes were further identified based on the criteria of module membership (|MM| > 0.8), gene significance (|GS| > 0.6), and *p* < 0.05. We identified the hub genes in the module that was most closely related to overfeeding-induced fatty liver and sex-related differences. The protein–protein interaction (PPI) network of hub genes was constructed using the STRING web server (https://cn.string-db.org/ accessed on 20 June 2025). Disconnected nodes were removed to focus on genes with direct or indirect interactions. The interaction confidence score was set to highest confidence (0.9), and multiple active interaction sources were enabled, including experiments, curated databases, co-expression, neighborhood, gene fusion, and co-occurrence. The Cytoscape (v3.9.1) [72] was used to map the gene–gene interaction network to visualize the gene relationships. Hub genes were ranked based on node degree.

### 4.8. RT-PCR Validation of AS Events

Total RNA purity and concentration were assessed using a NanoDrop spectrophotometer. For each sample, 1 μg of RNA was reverse-transcribed into cDNA using a reverse transcription kit (TIANGEN, Beijing, China). 35 cycles with annealing temperature of 58 °C for 45 s and elongation of 72 °C for 50 s were used in RT-PCR validation of AS events. 6 AS events were randomly selected from the DAS events (|IncLevelDifference| > 0.1, *p* < 0.05) for RT-PCR validation [14,34]. Primer sequences are listed in Table S1. PCR products were separated by 2% agarose gel in 50 × TAE buffer for 30 min at 140 V.

### 4.9. qPCR Validation of RNA-Seq Results

DEGs were randomly selected for validation by quantitative real-time PCR (qPCR) using the SuperReal PreMix Plus (SYBR Green) (TIANGEN, Beijing, China), following the manufacturer’s instructions. Data were analyzed for relative quantification using the 2^−ΔΔCT^ method [73], with *ACTB* as the reference gene. Primer specificity was evaluated by agarose gel electrophoresis, showing a predominant amplicon of the expected size and single-peak melting curves. The primers used for qPCR are listed in Table S2. qPCR experiments were performed with 6 biological replicates and 2 technical replicates. 6 DEGs were randomly selected for qPCR validation. Data analysis and visualization were conducted using GraphPad Prism (v9.5.0).

### 4.10. Statistical Analysis

Differences in liver phenotypic traits among groups were evaluated using one-way analysis of variance (ANOVA), and liver weight means and standard deviations were calculated using Excel (Office 2019, Microsoft Corporation, Redmond, WA, USA). Pearson correlation analysis was performed using GraphPad Prism to assess the consistency between RNA-seq and qRT-PCR results. The *p* values for DEGs and DAS events were adjusted for multiple testing using the Benjamini–Hochberg method.

## 5. Conclusions

This study provides an integrated transcriptomic and alternative splicing framework for understanding overfeeding-induced fatty liver formation in Lion-head geese. By combining differential expression analysis, WGCNA, and alternative splicing profiling, we identify both shared and sex-specific regulatory features underlying hepatic lipid accumulation. Notably, this work highlights alternative splicing as an additional regulatory layer linking transcriptional changes to intracellular remodeling during fatty liver development. Beyond its relevance to goose production, the Lion-head goose represents a well-established and physiologically tolerant model of hepatic steatosis, and our findings provide comparative insights into the molecular regulation of fatty liver that may be informative for studies of MASLD in other vertebrates. The hub genes and splicing-related candidates identified here, including *HYCC2*, *AGL*, *CCDC62*, *IGSF5*, *MGARP*, *CD80*, and *FPGS*, provide potential molecular markers and functional targets for future validation, which may support marker-assisted breeding and genetic improvement strategies aimed at optimizing fatty liver production while maintaining metabolic homeostasis.

## Data Availability

The data presented in this study are openly available in the Genome Sequence Archive (GSA) of the China National Center for Bioinformation (CNCB) at https://ngdc.cncb.ac.cn/gsa, reference number CRA032952. Accessed on 6 November 2025.

## Supplementary Materials

The following supporting information can be downloaded at: https://www.mdpi.com/article/10.3390/ijms27010407/s1.
