# Supplementary figures and images for: Weighted Gene Co-Expression Network Analysis and Alternative Splicing Analysis Reveal Key Genes Regulating Overfeeding-Induced Fatty Liver in Lion-Head Goose
Source: Int J Mol Sci. 2025 Dec 30;27(1):407. doi: 10.3390/ijms27010407 (PMC12785810; doi:10.3390/ijms27010407)

# Selection of Soft-Thresholding Power for WGCNA

## Scale independence

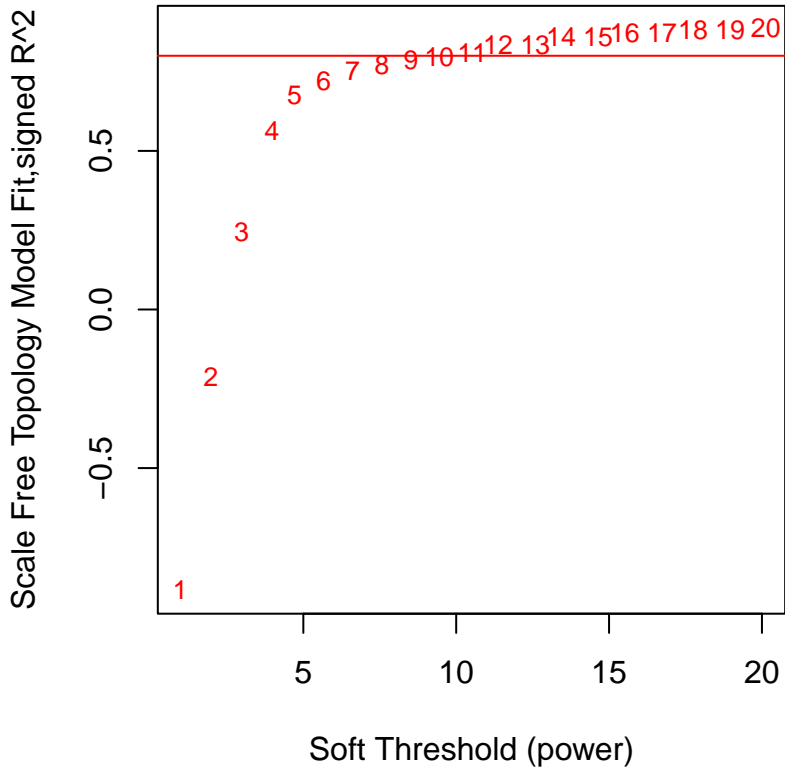

## Mean connectivity

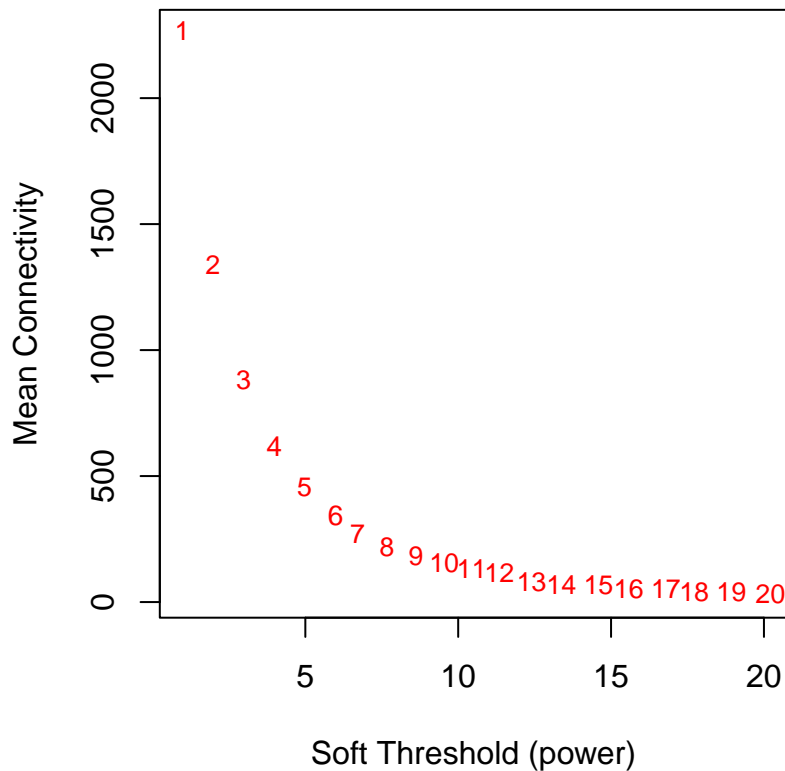

Supplement: Supplementary file 1 [file ijms-27-00407-s001.zip › Figture S1.pdf]
